# Shenqi Yanshen Formula (SQYSF) protects against chronic kidney disease by modulating gut microbiota

**DOI:** 10.1080/21655979.2021.2023789

**Published:** 2022-02-19

**Authors:** Ling Zhang, Tai-Jun Zhang, Ying Li, Wei-Jian Xiong

**Affiliations:** a Department of Nephrology, Chongqing Hospital of Traditional Chinese Medicine, Chongqing, Jiangbei, China; b The Office of Academic Affairs, Chongqing Hospital of Traditional Chinese Medicine, Chongqing, Jiangbei, China

**Keywords:** SQYSF, CKD, 16S, intestinal flora

## Abstract

In this study, we make an elucidation toward both the therapeutic effect and the mechanism of Shenqi Yanshen Formula (SQYSF) to chronic kidney disease (CKD). CKD mouse model was established and achieved in a way of adenine (200 mg/kg) perfusion. Six weeks later, those mice in the model group were fed with SQYSF (3.60 g/kg/day) every day (the captopril group was given 12.5 mg/kg/day by gavage every day, and control group and the model group were both given the gavage of equal volumes of normal saline); 4 weeks after the administration, we had our detection to physiological indicators of mice, performed ELISA assay to detect inflammatory factor expressions, then assay of 16S sequencing was used to reveal the difference of intestinal flora. Our results showed that after SQYSF treatment, both the expressions of serum creatinine (Scr) and blood urea nitrogen (BUN) came with a significant decline, indicating the outstanding performances of SQYSF in alleviating impairment in renal function and elevating mice’s physiological function. SQYSF significantly reduced the degree of renal fibrosis in CKD mice, and remarkably down-regulated the expressions of toll-like receptor 5 (TLR5), nuclear factor-kappaB (NF-κb), p65, tumor necrosis factor (TNF)-α, interleukin (IL)-1β and IL-6. Additionally, SQYSF has more than ability in significantly changing the composition in mice’s intestinal flora, but also in greatly increasing the abundance of Succinivibrionaceae and Aeromonadales in the mouse intestine. This study clarified the therapeutic effect of SQYSF on CKD and regulation of inflammatory factors and intestinal flora, and provided new ideas for treatment on CKD.

## Introduction

Chronic kidney disease (CKD) conventionally refers to the heterogeneous disorders impairing renal structure and function. Definition and classification of CKD continues to evolve and change over time; however, regardless of the root cause, current international guidelines define this condition as a decline in renal function, which is manifested as a glomerular filtration rate lower than 60 mL/min/1.73 m^2^, or a marker of kidney injury, or both, for at least 3 months [1]. In developed countries, CKD is usually associated with old age, diabetes, hypertension, obesity and cardiovascular disease [2]. CKD involves the accumulation of uremic toxins/metabolites, systemic inflammation and immune deficiency, and plays an important role in the pathogenesis of severe cardiovascular diseases and other CKD-related complications [3].

In the last decades, it was gradually realized that the profound impingement of microbiome is greatly associated with human physiology. The human gut is teeming with microbes, mainly anaerobic bacteria. These internalized ‘microbial organs’ are not coded in the host genome. They are made from at least 1013 dwellers and 500–1000 different species, and their collective genomes possibly have 100 times more genes than human own genome. Numerous evidence has shown that intestinal flora imbalance is associated with CKD [4,5]. Imbalance of the intestinal flora would produce excessive uremic toxins, and the damaged intestinal barrier fails to prevent these toxins from being transferred to the systemic circulation in this context [6,7]. It turns out that in many cases the accumulation of toxins in uremia is related to the progression of CKD risk.

According to improve the prognosis of Kidney Disease worldwide organization (Kidney diseases: Improving Global Outcomes, KDIGO) working group statistics, CKD is one of the most expensive chronic diseases and significantly reduce the patient’s life expectancy, although CKD5 needs dialysis and/or renal transplantation treatment period in patients with stage (i.e., uremia and end-stage CKD only 1%, but it consumes more than 5% of health budget) [8–10]. How to further delay the progression of the disease, extend the life span of the patients and achieve a higher quality of life is still an urgent problem to be solved. Clinical practices have shown that traditional Chinese medicine is effective in treating CKD. Chinese herbal medicine, the Shenqi Yanshen Formula (SQYSF), is composed of seven kinds of medicinal materials, red ginseng, astragalus root, raw rhubarb, herb epimedium, ligusticum wallichii, rehmannia root, vinegar-processed carapax trionycis, which is inherited from the experience of Professor Xin Zheng, a master of Chinese medicine, in treating chronic kidney disease. And it has achieved satisfactory results in many years of clinical treatment of CKD patients [11]. Chongqing Hospital of Traditional Chinese Medicine has developed SQYSF, which has the function of improving kidney function and eliminating toxins [11,12]. In recent years, modern medicine has also confirmed that Rehmannia glutinosa alleviates the level of renal interstitial fibrosis by down-regulating the levels of transforming growth factor (TGF)-β1, alpha-smooth muscle actin (α-SMA) and type I collagen [13,14].

Therefore, we established a CKD mouse model, where our evaluation to SQYSF’s efficiency on renal function and intestinal flora was carried out. We used network pharmacology tools in order to explore the therapeutic mechanism of SQYSF on CKD, and experimentally confirmed the fact that SQYSF reduces the expression of TLR5. We have designed the present study to verify the hypothesis of the good SQYSF therapeutic efficiency on CKD mouse model and the modulation on intestinal flora with the prospect of supporting the basic information of TCM, especially SQYSF in treating CKD patients.

## Materials and methods

### SQYSF

In this study, SQYSF consisted of seven natural herbs: red ginseng, astragalus root, raw rhubarb, herb epimedii, ligusticum wallichii, rehmannia root, vinegar-processed carapax trionycis (Table 1). SQYSF powder was prepared and standardized in Chongqing Institute of Traditional Chinese Medicine. The Chinese herbal medicines used in this study were prepared in accordance with the established guidelines of the 2010 edition of the Pharmacopoeia of the People’s Republic of China. Briefly, the herbs were boiled into decoction with water, filtering, concentrating, drying, pulverizing, and passing through a mesh to produce a powder for subsequent administration [15].

**Table 1.** SQYSF composition was listed by Chinese name, English name, Latin name; and each herb’s function was introduced

Chinese name	English name	Latin name	Function
Hongshen	Red ginseng	Ginsen Radix Et Rhizoma Rubra	Invigorating vitality, replenishing qi and invigorating blood
Huangqi	Astragalus root	Hedysarum Multijugum Maxim.	Enhancing the qi of spleen and kidney, improving renal function and pathological changes of kidney tissue [16]
Dahuang	Raw rhubarb	Radix Rhei Et Rhizome	Relieving diarrhea and turbidity and promoting blood circulation and removing blood stasis [17]
Yinyanghuo	Herb epimedium	Epimrdii Herba	Warming yang and nourishing the liver and kidney [18]
Chuanxiong	Ligusticum wallichii	Chuanxiong Rhizoma	Enriching blood and promoting blood circulation [19]
Shengdihuang	Rehmannia root	Rehmanniae Radix	Regulating immunity and delay kidney fibrosis [20,21]
Biejia	Vinegar-processed carapax trionycis	Trionycis Carapax	Anti-liver fibrosis, anti-cancer effects, and can enhance the immunity of experimental animals [22,23]

### Animal

C57BL/6 mice were obtained from the Animal Resource Center of Chongqing Medical University (Chongqing, China). Mice were given a standard diet, housed in a 12 h light/dark cycle. Mice with their weights of 16–20 g were utilized in all our experiments. All of the experiments were run in accordance with the guidelines for the care and use of experimental animals and the procedures for care and use of animals were approved by the Institutional Animal Care and Use Committee of Chongqing Medical University (Chongqing, China).

### Experimental design

The mice were kept under controlled humidity (55 + 15%) and temperature (23 + 2°C), 12 hours light/12 hours dark cycle. These mice were also allowed free access to standard laboratory food and water. Mice were randomized into 4 groups as follows:(1) control group; (2) CKD model group; (3) SQYSF treated group; (4) captopril treated group. CKD model group was given saline by intra-gastric gavage, SQYSF treated group was given SQYSF by intra-gastric gavage (3.6 g/kg/day); captopril treated group was given captopril by intra-gastric gavage (12.5 mg/kg/day) as positive control for its efficacy on CKD has been confirmed before [24–26]. Four weeks after the treatment, we collected blood and tissues for our next analysis. On the basis of previous clinical studies and standard conversion formulas below, we determined that the effective dose of SQYSF in mice was 3.6 g/kg/day [27].

$$Human\;equivalent\;dose(mg/kg)=Animal\left(mg/kg\right)\times {\left(Weight_{animal}\left[kg\right]/Weigtht_{human}\left[kg\right]\right)}^{\left(1-0.67\right)}$$

### Induction of chronic kidney disease

In this study, 48 C57BL/6 mice were selected, and after 7 days adaptive feeding, they were randomly divided into 12 blank groups and 36 model groups. Model group was given 2.5% adenine by intra-gastric gavage (250 mg/kg/day); blank groups were given equivalent saline by intra-gastric gavage. Two groups of mice were given continuous gavage 21 days once a day [28,29].

### Antibody

Antibody against TLR5 was purchased from Thermo Fisher (MA, USA). TNF-α, IL-1β, and IL-6 were purchased from Cell Signaling Technology (MA, USA). GAPDH was used as the antibodies reference in Western blotting analysis (1:1000 dilution, Abcam, UK). Goat anti-mouse secondary antibody and goat anti-rabbit secondary antibody were purchased from Santa Cruz Biotechnology (CA, USA).

### Histopathological staining analysis

In the histological analysis, we fixed the kidney tissue in 4% paraformaldehyde for at least 24 hours. After the tissues were sectioned (5 μm thick), embedded in paraffin. We used hematoxylin-eosin staining (HE) and Masson’s trichrome to observe the pathological changes and collagen deposition of kidney tissues [30].

### Assessment of kidney function

We used an intracardiac puncture method to take blood samples and centrifuged the samples at 3000 rpm for 15 minutes. The biochemical parameters of the kidney were obtained by measuring serum samples. The blood urea nitrogen and serum creatine levels were evaluated on the Hitachi 747 automatic analyzer (Hitachi Co., Ltd, Tokyo, Japan) platform.

### Western blotting

Cell lysis buffer (Beyotime Biotechnology, Jiangsu, China) was used to add 1 mM phenylmethanesulfonyl fluoride (Beyotime Biotechnology, Jiangsu, China) to extract protein lysates from kidney tissue. We used membrane and cytosolic protein extraction kit (Cat# P0033; Beyotime) to extract the cell membrane and cytoplasm. Equal amounts of protein samples were separated by 10% SDS/PAGE and transferred into polyvinylidene difluoride membranes (Millipore, Billerica, MA) [31]. The immunoblots were probed with primary antibodies (1:1000 dilution). Protein bands were visualized with Ultra-Sensitive Chemiluminescence kits (10,300, New Cell & Molecular Biotech Co., Ltd., Suzhou, China).

### Real-time PCR analysis

Specific TaqMan primers and probes for TLR5, TNF-α, IL-1β, IL-6 were obtained from Applied Biosystems. Specific TaqMan primers and probes for TLR5 (forward, 5ʹ-GCCCAGTGAGAACAGAAAGG-3ʹ, reverse,5ʹ-AAGGGAAAGGAAGGAAACAT-3ʹ), TNF-α(forward,5ʹ-AAGCCTGTAGCCCACGTCGTA-3ʹ, reverse,5ʹ-GGCACCACTAGTTGGTTGTCTTTG-3ʹ), IL-1β(forward,5ʹ-CTCTGACAGGCAACCACTTAC-3ʹ, reverse,5ʹ-GTCCAAATTCAATTCATCCC-3ʹ), IL-6(forward,5ʹ-TTGCCTTCTTGGGACTGATG-3ʹ, reverse,5ʹ-ACTGGTCTGTTGTGGGTGGT-3ʹ) were designed using Primer Express Software (Applied Biosystems). According to the manufacturer’s instructions, we used gene-specific primers and probes on the ABI Prism 7700 Sequence Detection System (Applied Biological System) to amplify cDNA in 1 × Universal Master Mix (Applied Biological System). The PCR condition was set to: 94°C for 30 s, 55°C for 30 s, 72°C for 30 s, 35 cycles. Our analysis of the data obtained was commenced by Sequence Detector V1.9 analysis software (Applied Biosystems) [32]. The expression of each gene was standardized with the mRNA expression of the housekeeping gene β-actin.

### ELISA

TNF-α, TGF-β, and TLR5 in serum were measured using the specific ELISA kits (R&D Co., Ltd, MN, USA) according to the manufacturer's protocol. In short, the supernatant or controls were added to a 96-well plate coated with coated antibodies, which was then incubated at room temperature for 2 hours. Plates were washed five times, after that a detecting antibody was added to each well. Plates were incubated at room temperature for 2 h and 100 μL solution was added to each well. After incubation for 90 min, 100 μL stop solution was added to each well. The absorbance was measured at 450 nm [32].

### DNA extraction

This section contains 16S Ribosomal RNA (rRNA) Gene Sequencing, and Microbial Analysis of Fecal Samples. Fecal samples were frozen at −80°C for 3 hours after collection. QIAamp Fast DNA Stool Mini Kit (Qiagen, California, USA) was used to extract DNA samples. We then determined the purity of the DNA samples and calculated their concentration. Bacterial 16S rRNA gene V3 region was amplified by using PCR. The bacterial genomic DNA was amplified by PCR with forward primer (5-TCGTCGGCAGCGTCAGATGTGTATAAGAGACAGCCTACGGGNGGCWGCAG) and reverse primer (5-GTCTCGTGGGCTCGGAGATGTGTATAAGAGACAGGACTACHVGGGTATCTAATCC) for the V3 hypervariable region. Purified amplicons were pooled into equimolar concentrations, and paired end sequencing was performed using an Illumina MiSeq instrument (Illumina, San Diego, California, USA). Representative sequences of OTUs are used to analyze diversity based on their relative abundance (Chao index and Shannon diversity index). R software was used to generate heat maps based on the relative abundance of OTUs (https://www.R-project.org). We used the representative sequence of each sample OTUs to measure the phylogenetic diversities, such as unweighted UniFrac significance test, principal coordinate analysis and non-metric multidimensional scale analysis to analyze the community and phylogeny. We used a ribosomal database project classifier with a 60% bootstrap score to perform taxonomy-based analyses on taxonomic classification.

### Network pharmacology

On the basis of the network pharmacology principles, the main target of Shenqi Yanshen Formula is predicted, paving the way for further experiments for exploring its mechanism of action. Retrieve the effective active ingredients and relevant targets of Shenqi Yanshen Formula from TCMSP (https://tcmspw.com/tcmsp.php), and select chronic kidney disease according to the Genecards (https://www.genecards.org/) disease database (CKD) related genes, upload the obtained common target to the String website (https://string-db.org/) to obtain the protein–protein interaction network (PPI); upload the core target to DAVID (https://david.ncifcrf.gov/home.jsp), limited species ‘Homo sapiens’, use the drawing software Cytoscape 3.6.1 to analyze the GO biological process and KEGG pathway enrichment analysis of key targets [33].

### Statistical analysis

All of our data were shown in a manner of mean + standard deviation. We had our statistical analysis with the help of SPSS 13.0 software. Analyses of the statistical distinctions amid the experiment and the control groups were accomplished by the method of least significant difference (LSD); method of t-test was used for two groups, whereas that of one-way analysis of variance (ANOVA) for multiple groups. P values <0.05 were deemed statistically significant.

## Result

Traditional Chinese medicine treatment has unique advantages in delaying the progression of CKD and improving the quality of life, especially for the prevention and treatment of chronic kidney disease stage 3–4. Due to its multi-target and synergistic characteristics, traditional Chinese medicine can delay the progression of renal failure, reduce complications, and delay the entry of renal replacement therapy. In this study, a CKD mouse model was induced by adenine (200 mg/kg), and the therapeutic effect and mechanism of SQYSF on CKD were detected and analyzed. ELISA was used to detect the expression of inflammatory factors, 16S sequencing was used to reveal the difference of intestinal flora, and the effect of SQYSF on kidney fibrosis in CKD mice was also tested. As a traditional Chinese medicine, SQYSF has the effects of invigorating the kidney, promoting blood circulation, detoxification and reducing turbidity, and could exhibit a significant therapeutic effect in the CKD mouse model.

### SQYSF alleviated renal functional damage in CKD mice

**Figure 1.**
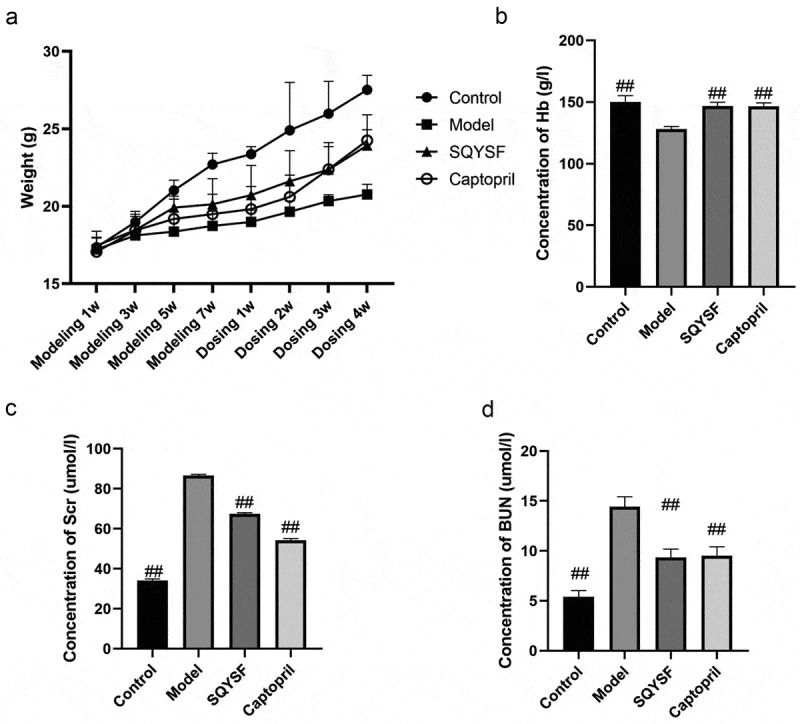
SQYSF alleviated renal functional damage in CKD mice. (a) The body weights of modeling 1 week, 3 weeks, 5 weeks, 7 weeks and 1 week, 2 weeks, 3 weeks and 4 weeks after administration were measured respectively. (b) Detect the Hb content after 4 weeks of administration. (c) Detect the Scr content after 4 weeks of administration. (d) Detect the BUN content after 4 weeks of administration. ##, p < 0.01, compared with the model group.

In order to study the effect of SQYSF on the renal function of CKD mice, we detected the body weight, hemoglobin (Hb), serum creatinine (Scr) and blood urea nitrogen (BUN) of each mouse. Compared with model group, SQYSF group and Captopril group, body weight and hemoglobin increased significantly, and SQYSF significantly improved the physiological functions of mice (Figure 1(a,b)). Compared with control group, the expressions of Scr and BUN in model group increased significantly, indicating that the renal function of CKD was severely decreased. Compared with model group, SQYSF group and Captopril group, expressions of Scr and BUN decreased significantly, indicating that SQYSF can effectively alleviate the damage to renal function. There is no significant difference between SQYSF group and Captopril group (Figure 1(c,d)).

### SQYSF alleviated renal inflammation and fibrosis in CKD mice

**Figure 2.**
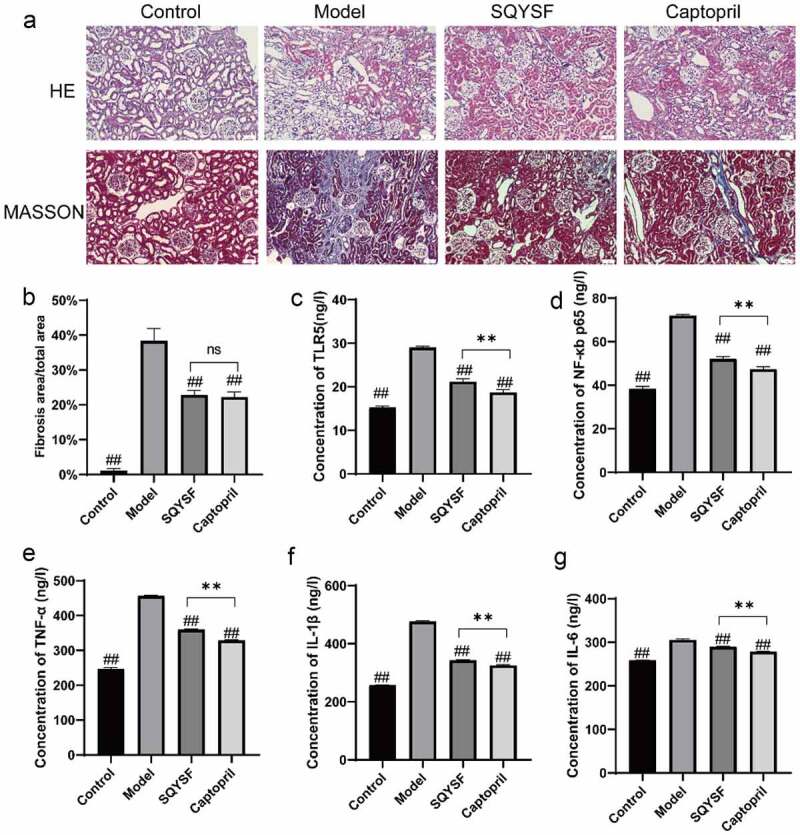
SQYSF alleviated renal inflammation and fibrosis in CKD mice. (a) HE staining to detect kidney morphology in each group, Masson staining to detect kidney fibrosis in each group. (b) Masson staining statistical results. (c–g) ELISA to detect the contents of TLR5, NF-κb, p65, TNF-α, IL-1β and IL-6 in each group of serum samples. ##, p < 0.01, which were compared with model group. ns, p > 0.05, **, p < 0.01, SQYSF compared with captopril group.

To investigate the effects of SQYSF on inflammation and fibrosis in CKD mice, we observed the changes of renal tissue structure in four groups of mice with HE staining and Masson staining, and detected the level of TLR5, NF-κb p65, TNF-α, IL-1β and IL-6 in mice. Outcomes of HE staining revealed that control group was normal renal tissue, and the model group mice were glomerulosclerosis, renal tubular dilatation, epithelial cell necrosis and inflammatory cell infiltration (Figure 2(a)). The results of Masson staining showed that large area of blue stained collagen fiber was found in model group, and the area of fibrosis in SQYSF group and captopril group was significantly lower than that in the same group and moreover no significant difference between SQYSF group and captopril group (Figure 2(a,b)). When compared with model group, the level of TLR5, NF-κb p65, TNF-α, IL-1β and IL-6 down-regulated significantly in SQYSF group and captopril group, while it does not totally restore as the controls (Figure 2(c,g)). Collectively, SQYSF alleviated renal inflammation and fibrosis in CKD mice.

### The core components and mechanism of SQYSF was investigated in the treatment of CKD by using network pharmacology

**Figure 3.**
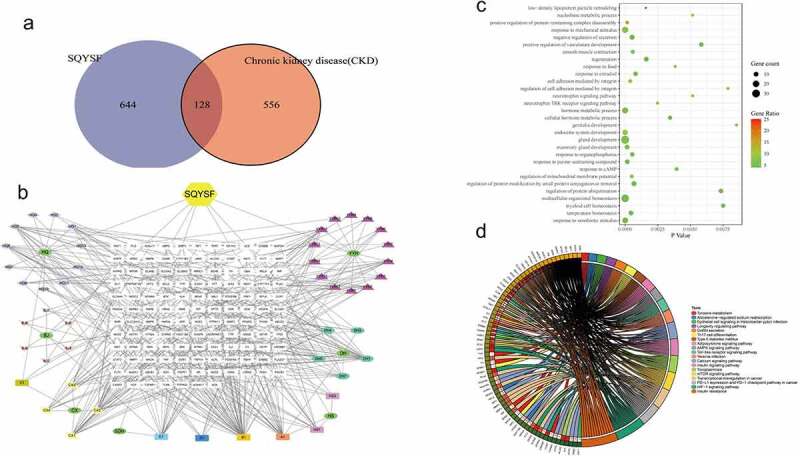
Network pharmacological analysis of SQYSF. (a) SQYSF and CKD target intersection gene Venn diagram. (b) drug-active ingredient-target network diagram. SQYSF represents the drug Shenqi Yanshen Formula; HQ, BJ, CX, SDH, HS, DH, and YYH represent the 7 single-drugs of Shenqi Yanshen Formula: Astragalus, Biejia, Chuanxiong, Rehmannia, Red Ginseng, Rhubarb and Yin Sheep Huo. (c) Intersection gene GO analysis BP bubble chart. The Y-axis on the left is the name of the GO pathway, and the abscissa is the p value. (d) Signal pathway enrichment map. The larger the circle, the more the number of genes compared to the pathway, and the darker the color indicates the higher the proportion of the compared genes in the pathway. KEGG pathway enrichment analysis circle picture. The right side of the outermost circle is the name of the signal pathway, and the left side is the gene. The inner circle on the left indicates the significance p value of the corresponding pathway of the gene.

After network pharmacological analysis, it was found that SQYSF has a total of 772 molecular targets, of which 684 are related to CKD, and there are 128 intersecting targets between the two (Figure 3(a)). The relationship network of SQYSF-monomer-target is shown in Figure 3(b). Through GO analysis, it is found that SQYSF has a greater impact on low-density lipoprotein particle remodeling and positive regulation of protein-containing complex disassembly in the biological process (Figure 3(c)). The results showed that the relevant pathways of SQYSF in the treatment of CKD include Tyrosine metabolism, Calcium signaling pathway, mTOR signaling pathway, AMPK signaling pathway, PD-L1 expression and PD-1 checkpoint pathway in cancer, Toll-like receptor signaling pathway, HIF-1 signaling pathway. By analyzing the component-target map and KEGG pathway, we believe that the main mechanism of SQYSF treatment may be that the kaempferol, eupatin, beta-sitosterol, and myricanone components act on HIF-1 signaling pathway, mTOR signaling pathway, Toll-like receptor signaling pathway, indicating the treatment characteristics of SQYSF with multiple components, multiple targets and multiple pathways (Figure 3(d)).

### SQYSF inhibited the TLR5 Signaling Pathway in the kidney of CKD mice

**Figure 4.**
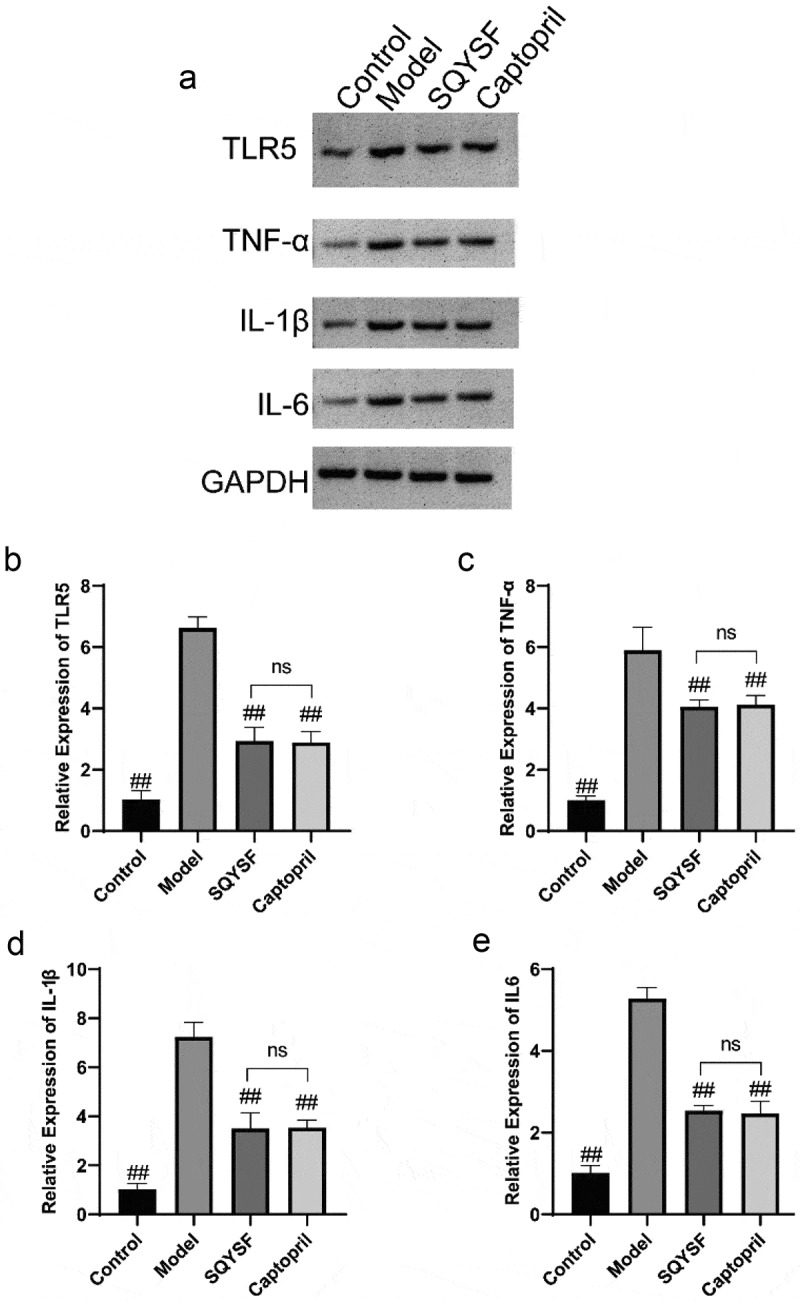
SQYSF inhibited the TLR5 Signaling Pathway in the kidney of CKD mice. (a) Western blot detection on the protein expressions of TLR5 and TNF-α, IL-1β and IL-6. (b–e) qPCR detection on the mRNA expressions of TLR5 and TNF-α, IL-1β and IL-6. ##, p < 0.01, which was compared with model group. ns, p > 0.05, SQYSF compared with captopril group.

Western blot and qPCR results showed that TLR5 expression level and TNF-α, IL-1β and IL-6 levels in model group were greatly up-regulated compared with control group, indicating activation of TLR5 signaling pathway in CKD mice. TLR5, TNF-α, IL-1β and IL-6 levels were significantly decreased in SQYSF group and Captopril group compared with model group. And SQYSF group has a comparable effect compared with Captopril group (Figure 4), suggesting that the TLR5 signaling pathway was inhibited in SQYSF and Captopril treated CKD mice.

### SQYSF therapeutic effects on the Gut Microbiota Composition of CKD mice

**Figure 5.**
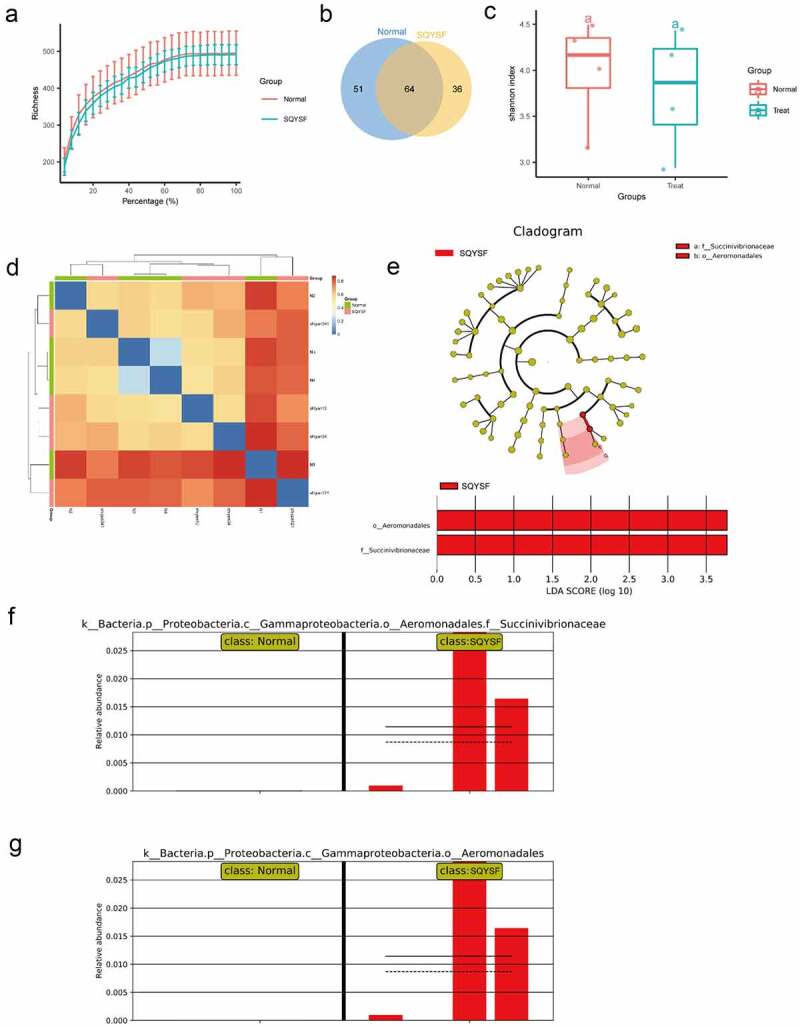
16s sequencing analysis of SQYSF’s regulatory effect on mouse intestinal flora. (a) Sequencing data abundance of normal group and SQYSF group. (b) Venn diagram shows the difference in flora between the two groups. (c) α-diversity of gut microbiota (Shannon index) and (d) β-diversity of gut microbiota using Bray-Curtis distance. (e) LEfSe analysis estimates the influence of the abundance of different strains on the difference effect. (f) relative Succinivibrionaceae abundance in two groups. (g) relative abundance of Aeromonadales in the two groups.

Next, in order to clarify the effect of SQYSF on the intestinal flora after 4 weeks of treatment of CKD mice. We collected the feces in the intestines of mice in the normal and the SQYSF groups for 16S DNA sequencing. The abundance of the sequencing data for the two sets of samples is shown in Figure 5(a). According to the OTU analysis of the sequencing data, 51 species were significantly expressed in the normal group, and 36 species were notably expressed in the SQYSF group (Figure 5(b)). Then, α-diversity and β-diversity of gut microbiota analysis in normal and SQYSF groups was performed. After SQYSF treatment, the Shannon index decreased, and the species diversity of the flora reduced (Figure 5(c)). And β-diversity analysis showed SQYSF treatment affected the composition of the fecal gut microbiota (Figure 5(d)). Furthermore, we use LDA Effect Size (LEfSe analysis) to estimate the size of the influence that different strains’ abundance on the difference effect, and find the colonies that have a significant difference in the sample division. It was found that after SQYSF treatment, f_Succinivibrionaceae and o_Aeromonadales were the most significant. It indicates that these two strains are likely to participate in the regulation pathway of CKD (Figure 5(e–g)).

## Discussion

CKD is a disease that changes the structure and function of the kidney, and is a typical predictor of end-stage kidney disease [34]. In developed countries, chronic kidney disease is usually closely related to cardiovascular disease, diabetes, hypertension, and obesity, but the mechanism of CKD has not yet been clearly studied [35,36]. In this study, the CKD mouse model was constructed, and SQYSF was added to detect the protective effect of SQYSF on the kidneys of CKD mice, the expression of inflammatory factors, and the difference of intestinal flora. The results show that SQYSF can effectively reduce renal fibrosis in CKD mice, significantly reduce the expression of inflammatory factors TNF-α, IL-1β and IL-6, and can significantly change the composition of the mouse intestinal flora. And we found that SQYSF significantly increase the abundance of f_Succinivibrionaceae and o_Aeromonadales in the mouse intestine. This study preliminarily analyzed the therapeutic effect of SQYSF on CKD and some molecular mechanisms, and provided new ideas for better treatment of CKD.

The current mainstream view is that CKD is related to uremic toxin/metabolite accumulation, systemic inflammation and immune deficiency [37,38]. Recent studies pointed out that the intestinal microbiota plays a key role in symptoms, such as chronic inflammation [39–41]. In the digestive tract of healthy people, there are a large number of bacteria. The density of these bacteria in the ileum reaches 10^6^–10^8^ /mL, and the density in the colon is as high as 10^12^/mL. Among these intestinal bacteria, more than 90% of bacteria can be classified into the phylum Bacteroides and Hard-walled bacteria [42,43]. There are both ‘probiotics’ that are beneficial to the body like bifidobacteria, and there are also a large number of ‘saprobiotics’ that are harmful to the body [44–46]. The gut microbiome of CKD was characterized by Zhigang Ren et al., and was classified by microbial markers. It was found that compared with healthy controls, CKD gut microbial diversity was significantly reduced, and the microbial community is distinct to HC [6]. Also, several studies have indicated the changes in the quantification and quality of gut microbiota in patients with CKD [47]. A large number of studies have found that intestinal microbes mainly affect CKD through different pathways through uremic toxins (indoxyl sulfate, sulfuric acid, and trimethylamine-N-oxide) [48–50]. The hormone-angiotensin-aldosterone system or the mechanism that promotes oxidative stress aggravates the kidney damage in CKD patients. In this study, after SQYSF treated CKD mice for 4 weeks, the stools in the intestine were collected for 16S DNA sequencing. In the normal group, 51 species were significantly expressed, and 36 species were significantly expressed in the SQYSF group. And we found that after SQYSF treatment, f_Succinivibrionaceae and o_Aeromonadales were the most significant. It indicates that these two strains are likely to participate in the regulation of CKD. Studies have compared the difference in intestinal flora between non-survivor and survivor with CKD, and found that the abundance of Succinivibrionaceae and Anaerostipes producing short-chain fatty acid is higher in the survivor group, which has been shown to have a wide range of effects on host physiology, including anti-inflammatory effects and maintaining intestinal integrity [51]. Aeromonadales is classified as aerobic and facultative anaerobes, which can cause many diseases such as enteritis and sepsis, and is also related to kidney and cardiovascular problems [52,53].

CKD would cause the imbalance within different intestinal floras and damage the intestinal epithelial barrier by destroying the tight junctions of the colonic epithelium and reducing the survival rate of the epithelium [54,55]. Loss of the integrity of the intestinal epithelial barrier leads to bacterial and lipopolysaccharide translocations, disrupts immune responses, and inflammatory responses. Lipopolysaccharide can activate natural killer cells by activating TLR5-dependent and NF-KB pathways [56]. Pathogenic bacteria stimulate dendritic cells to activate Th17/Th1 T cells, increase the production of inflammatory factors, carbohydrates, bile acids and proteins in the intestine are fermented by intestinal pathogenic bacteria to produce indoxyl sulfate, trimethylamine-N-oxide and other harmful substances [57,58]. A decrease in probiotics, particularly bifidobacteria, leads to a decrease in short-chain fatty acids. Gram-negative bacilli in the intestine proliferate in large numbers produce a large amount of endotoxin, and enter the blood through the intestinal epithelial barrier, relying on TLR5 to activate various downstream inflammatory cascades. In this study, in an experiment to study the effect of SQYSF on inflammation and fibrosis in CKD mice, HE staining and Masson staining showed that after SQYSF treatment, the area of kidney fibrosis in CKD mice was significantly reduced, and the content of inflammatory factors was significantly reduced. In addition, after SQYSF treatment, the levels of Scr and BUN decreased significantly, indicating that SQYSF can effectively reduce the damage to renal function and significantly improve the physiological functions of mice.

Our study had other analyses to SQYSF network pharmacology, and then the outcomes pinpointed those pathways relevant to SQYSF within CKD treatment, which are the signaling pathways of Tyrosine metabolism, Calcium, mTOR, AMPK, Toll-like receptor, HIF-1 and the pathways of the PD-L1 expression and PD-1 checkpoint in cancer. By analyzing the component-target map and KEGG pathway, we believe that the pharmacological mechanism of SQYSF is mainly accomplished through the efficiency of kaempferol, eupatin, beta-sitosterol, and myricanone components onto the signaling pathways of HIF-1, mTOR, Toll-like receptor, indicating the characteristics of SQYSF’s multi-component, multi-target, and multi-channel treatment. Through GO analysis, it is found that SQYSF has a greater impact on low-density lipoprotein particle remodeling and positive regulation of protein-containing complex disassembly in the biological process. Of course, this study fails to include all the many databases in the statistical scope, which may lead to certain limitations in the prediction results, which still need to be further verified by basic research. The joint analysis of network pharmacology, intestinal flora, metabolome, and transcriptome, and drawing a more comprehensive and in-depth network of SQYSF regulating CKD will be the direction of our next research.

## Conclusion

SQYSF has a significant therapeutic effect on renal fibrosis in mice, which is manifested in the significant inhibition to the expression of inflammatory factors (TNF-α, IL-1β and IL-6), in addition to significantly changing mice’s composition intestinal flora; moreover, SQYSF is capable of greatly improving the abundance of Succinivibrionaceae and Aeromonadales in the mouse intestine.

## Highlights

- SQYSF alleviates the impairment in renal function and renal fibrosis in CKD mice.
- SQYSF decreases serum inflammation factors expression in CKD mice.
- SQYSF can change the composition of gut microbiota in CKD mice.

## References

[1] Qin Y, Tang H, Yan G, et al. A high triglyceride-glucose index is associated with contrast-induced acute kidney injury in Chinese patients with type 2 diabetes mellitus. Front Endocrinol (Lausanne). 2020;11:522883. 33551987 10.3389/fendo.2020.522883 PMC7862330

[2] Heerspink HJL, Stefánsson BV, Correa-Rotter R, et al. Dapagliflozin in patients with chronic kidney disease. N Engl J Med. 2020;383(15):1436–1446. 32970396 10.1056/NEJMoa2024816

[3] McCormick N, Zhang Y, Choi HK. Allopurinol and chronic kidney disease. N Engl J Med. 2020;383(17):1689–1690. 10.1056/NEJMc2026125 33085874

[4] Moraes C, Borges NA, Mafra D. Resistant starch for modulation of gut microbiota: promising adjuvant therapy for chronic kidney disease patients? Eur J Nutr. 2016;55(5):1813–1821. 26830416 10.1007/s00394-015-1138-0

[5] Esgalhado M, Borges NA, Mafra D. Could physical exercise help modulate the gut microbiota in chronic kidney disease? Future Microbiol. 2016;11:699–707. 27159232 10.2217/fmb.16.12

[6] Ren Z, Fan Y, Li A, et al. Alterations of the human gut microbiome in chronic kidney disease. Adv Sci (Weinh). 2020;7(20):2001936. 33101877 10.1002/advs.202001936 PMC7578882

[7] Armani RG, Ramezani A, Yasir A, et al. Gut microbiome in chronic kidney disease. Curr Hypertens Rep. 2017;19(4):29. 28343357 10.1007/s11906-017-0727-0

[8] End chronic kidney disease neglect. Nature. 2020;579(7798):173. 10.1038/d41586-020-00691-4 32161393

[9] Webster AC, Nagler EV, Morton RL, et al. Chronic kidney disease. Lancet. 2017;389(10075):1238–1252. 27887750 10.1016/S0140-6736(16)32064-5

[10] Allison SJ. Finerenone in chronic kidney disease. Nat Rev Nephrol. 2021;17(1):13. 33110253 10.1038/s41581-020-00371-6

[11] Ying L, Tai-jun Z, Hong L, et al. Clinical observation on patients with chronic kidney disease stage 3-5 treated by the Chinese herbs formula of shenqiyanshenfang. China Med Guide. 2019;17(13):1–2.

[12] Bo L. Observation on the effect of shenqi yanshen decoction in adjuvant treatment of early chronic kidney disease. China Rural Med. 2021;28(6):45–46.

[13] G LD, M Z, Y GH. Rehmanniae radix and rehmanniae radix praeparata ameliorates renal interstitial fibrosis induced by unilateral ureteral occlusion in rats and their mechanism. Zhong Yao Cai. 2015;38(12):2507–2510. 27352530

[14] Zeng O, Li F, Li Y, et al. Effect of novel gasotransmitter hydrogen sulfide on renal fibrosis and connexins expression in diabetic rats. Bioengineered. 2016;7(5):314–320. 27575818 10.1080/21655979.2016.1197743 PMC5060972

[15] Xuan Y, Ding D, Xuan W, et al. A traditional Chinese medicine compound (Jian Er) for presbycusis in a mouse model: reduction of apoptosis and protection of cochlear sensorineural cells and hearing. Int J Herb Med. 2018;6(6):127–135. 31890893 PMC6936738

[16] Liao H, Hu L, Cheng X, et al. Are the therapeutic effects of Huangqi (Astragalus membranaceus) on diabetic nephropathy correlated with its regulation of macrophage iNOS activity? J Immunol Res. 2017;2017:3780572. 29250558 10.1155/2017/3780572 PMC5698796

[17] Sham TT, Chan CO, Wang YH, et al. A review on the traditional Chinese medicinal herbs and formulae with hypolipidemic effect. Biomed Res Int. 2014;2014:925302. 25110708 10.1155/2014/925302 PMC4109135

[18] Wu J, Li W, Ye B, et al. The efficacy and safety of Xianling Gubao capsules in the treatment of knee osteoarthritis: a protocol for a randomized, double-blind, controlled trial. Medicine (Baltimore). 2021;100(36):e27086. 34516497 10.1097/MD.0000000000027086 PMC8428714

[19] Guo M, Liu Y, Shi D. Cardiovascular actions and therapeutic potential of tetramethylpyrazine (Active component isolated from rhizoma chuanxiong): roles and mechanisms. Biomed Res Int. 2016;2016:2430329. 27314011 10.1155/2016/2430329 PMC4893570

[20] Hu Z, Gao L, Li C, et al. Efficacy of Longdan Xiegan decoction on the treatment of eczema: a systematic review and meta-analysis. Evid Based Complement Alternat Med. 2021;2021:8836117. 33680065 10.1155/2021/8836117 PMC7906809

[21] Lee BC, Choi JB, Cho HJ, et al. Rehmannia glutinosa ameliorates the progressive renal failure induced by 5/6 nephrectomy. J Ethnopharmacol. 2009;122(1):131–135. 19146934 10.1016/j.jep.2008.12.015

[22] Dong Q, Qiu LL, Zhang CE, et al. Identification of compounds in an anti-fibrosis Chinese medicine (Fufang Biejia Ruangan Pill) and its absorbed components in rat biofluids and liver by UPLC-MS. J Chromatogr B Analyt Technol Biomed Life Sci. 2016;1026:145–151. 10.1016/j.jchromb.2015.12.024 26724854

[23] Zhang Y, Mao X, Chen W, et al. A discovery of clinically approved formula FBRP for repositioning to treat HCC by inhibiting PI3K/AKT/NF-κB activation. Mol Ther Nucleic Acids. 2020;19:890–904. 31982775 10.1016/j.omtn.2019.12.023 PMC6994416

[24] Chan GCW, Wu HJ, Chan KW, et al. N-acetyl-seryl-aspartyl-lysyl-proline mediates the anti-fibrotic properties of captopril in unilateral ureteric obstructed BALB/C mice. Nephrology (Carlton). 2018;23(4):297–307. 28075040 10.1111/nep.13000

[25] Rasool MF, Ali S, Khalid S, et al. Development and evaluation of physiologically based pharmacokinetic drug-disease models for predicting captopril pharmacokinetics in chronic diseases. Sci Rep. 2021;11(1):8589. 33883647 10.1038/s41598-021-88154-2 PMC8060346

[26] Sonfack CS, Nguelefack-Mbuyo EP, Kojom JJ, et al. The aqueous extract from the stem bark of garcinia lucida vesque (Clusiaceae) exhibits cardioprotective and nephroprotective effects in adenine-induced chronic kidney disease in rats. Evid Based Complement Alternat Med. 2021;2021:5581041. 33790975 10.1155/2021/5581041 PMC7984895

[27] Nair AB, Jacob S. A simple practice guide for dose conversion between animals and human. J Basic Clin Pharm. 2016;7(2):27–31. 27057123 10.4103/0976-0105.177703 PMC4804402

[28] Mandai S, Mori T, Nomura N, et al. WNK1 regulates skeletal muscle cell hypertrophy by modulating the nuclear localization and transcriptional activity of FOXO4. Sci Rep. 2018;8(1):9101. 29904119 10.1038/s41598-018-27414-0 PMC6002401

[29] Mazumder MK, Paul R, Bhattacharya P, et al. Neurological sequel of chronic kidney disease: from diminished Acetylcholinesterase activity to mitochondrial dysfunctions, oxidative stress and inflammation in mice brain. Sci Rep. 2019;9(1):3097. 30816118 10.1038/s41598-018-37935-3 PMC6395638

[30] Xu Z, Zhao Y, Zhong P, et al. EGFR inhibition attenuates diabetic nephropathy through decreasing ROS and endoplasmic reticulum stress. Oncotarget. 2017;8(20):32655–32667. 28427241 10.18632/oncotarget.15948 PMC5464817

[31] Yao H, Chi X, Jin Y, et al. Dexmedetomidine inhibits TLR4/NF-κB activation and reduces acute kidney injury after orthotopic autologous liver transplantation in rats. Sci Rep. 2015;5:16849. 26585410 10.1038/srep16849 PMC4653646

[32] Pereira NS, Queiroga TBD, Nunes DF, et al. Innate immune receptors over expression correlate with chronic chagasic cardiomyopathy and digestive damage in patients. PLoS Negl Trop Dis. 2018;12(7):e0006589. 30044791 10.1371/journal.pntd.0006589 PMC6078325

[33] An L, Feng F. Network pharmacology-based antioxidant effect study of zhi-zi-da-huang decoction for alcoholic liver disease. Evid Based Complement Alternat Med. 2015;2015:492470. 25922610 10.1155/2015/492470 PMC4398926

[34] Hobby GP, Karaduta O, Dusio GF, et al. Chronic kidney disease and the gut microbiome. Am J Physiol Renal Physiol. 2019;316(6):F1211–f1217. 30864840 10.1152/ajprenal.00298.2018 PMC6620595

[35] Meijers B, Evenepoel P, Anders HJ. Intestinal microbiome and fitness in kidney disease. Nat Rev Nephrol. 2019;15(9):531–545. 31243394 10.1038/s41581-019-0172-1

[36] Tang WHW, Li DY, Hazen SL. Dietary metabolism, the gut microbiome, and heart failure. Nat Rev Cardiol. 2019;16(3):137–154. 30410105 10.1038/s41569-018-0108-7 PMC6377322

[37] Felizardo RJF, Watanabe IKM, Dardi P, et al. The interplay among gut microbiota, hypertension and kidney diseases: the role of short-chain fatty acids. Pharmacol Res. 2019;141:366–377. 30639376 10.1016/j.phrs.2019.01.019

[38] Sueyoshi M, Fukunaga M, Mei M, et al. Effects of lactulose on renal function and gut microbiota in adenine-induced chronic kidney disease rats. Clin Exp Nephrol. 2019;23(7):908–919. 30895529 10.1007/s10157-019-01727-4 PMC6555783

[39] Meijers B, Farré R, Dejongh S, et al. Intestinal barrier function in chronic kidney disease. Toxins (Basel). 2018;10(7):298. 10.3390/toxins10070298 PMC6071212 30029474

[40] Sircana A, De Michieli F, Parente R, et al. Gut microbiota, hypertension and chronic kidney disease: recent advances. Pharmacol Res. 2019;144:390–408. 29378252 10.1016/j.phrs.2018.01.013

[41] Ruszkowski J, Witkowski JM. Lactulose: patient- and dose-dependent prebiotic properties in humans. Anaerobe. 2019;59:100–106. 31176002 10.1016/j.anaerobe.2019.06.002

[42] Arias N, Arboleya S, Allison J, et al. The relationship between choline bioavailability from diet, intestinal microbiota composition, and its modulation of human diseases. Nutrients. 2020;12(8):2340. 10.3390/nu12082340 PMC7468957 32764281

[43] Glorieux G, Gryp T, Perna A. Gut-derived metabolites and their role in immune dysfunction in chronic kidney disease. Toxins (Basel). 2020;12(4):245. 10.3390/toxins12040245 PMC7232434 32290429

[44] Hu Q, Wu K, Pan W, et al. Intestinal flora alterations in patients with early chronic kidney disease: a case-control study among the Han population in southwestern China. J Int Med Res. 2020;48(6):300060520926033. 32495708 10.1177/0300060520926033 PMC7273791

[45] Konrad L, Andersen K, Kesper MS, et al. The gut flora modulates intestinal barrier integrity but not progression of chronic kidney disease in hyperoxaluria-related nephrocalcinosis. Nephrol Dialysis Transplantation. 2019;35(1):86–97. 10.1093/ndt/gfz080 31081025

[46] Jiang S, Wang B, Sha T, et al. Changes in the intestinal microbiota in patients with stage 5 chronic kidney disease on a low-protein diet and the effects of human to rat fecal microbiota transplantation. Med Sci Monit. 2020;26:e921557. 32592577 10.12659/MSM.921557 PMC7336834

[47] Hanifi GR, Samadi Kafil H, Tayebi Khosroshahi H, et al. Bifidobacteriaceae family diversity in gut microbiota of patients with renal failure. Arch Razi Inst. 2021;76(3):521–528. 34824745 10.22092/ari.2020.352271.1557 PMC8605840

[48] Wu IW, Lin CY, Chang LC, et al. Gut microbiota as diagnostic tools for mirroring disease progression and circulating nephrotoxin levels in chronic kidney disease: discovery and validation study. Int J Biol Sci. 2020;16(3):420–434. 32015679 10.7150/ijbs.37421 PMC6990903

[49] He LX, Abdolmaleky HM, Yin S, et al. Dietary fermented soy extract and oligo-lactic acid alleviate chronic kidney disease in mice via inhibition of inflammation and modulation of gut microbiota. Nutrients. 2020;12(8):2376. 10.3390/nu12082376 PMC7468970 32784477

[50] Lun H, Yang W, Zhao S, et al. Altered gut microbiota and microbial biomarkers associated with chronic kidney disease. Microbiologyopen. 2019;8(4):e00678. 30088332 10.1002/mbo3.678 PMC6460263

[51] Lin TY, Wu PH, Lin YT, et al. Gut dysbiosis and mortality in hemodialysis patients. NPJ Biofilms Microbiomes. 2021;7(1):20. 33658514 10.1038/s41522-021-00191-x PMC7930281

[52] Bhowmick UD, Bhattacharjee S. Bacteriological, clinical and virulence aspects of aeromonas-associated diseases in humans. Pol J Microbiol. 2018;67(2):137–149. 30015452 10.21307/pjm-2018-020 PMC7256846

[53] Wang Q, Liu K, Jin C. Clinical value of microRNA-378a-3p in sepsis and its role in sepsis-induced inflammation and cardiac dysfunction. Bioengineered. 2021;12(1):8496–8504. 34565302 10.1080/21655979.2021.1985339 PMC8806767

[54] Lee TH, Park D, Kim YJ, et al. Lactobacillus salivarius BP121 prevents cisplatin induced acute kidney injury by inhibition of uremic toxins such as indoxyl sulfate and pcresol sulfate via alleviating dysbiosis. Int J Mol Med. 2020;45(4):1130–1140. 32124946 10.3892/ijmm.2020.4495 PMC7053870

[55] Lakshmanan AP, Al Za’abi M, Ali BH, et al. The influence of the prebiotic gum acacia on the intestinal microbiome composition in rats with experimental chronic kidney disease. Biomed Pharmacother. 2021;133:110992. 33202283 10.1016/j.biopha.2020.110992

[56] Perumal Samy R, Stiles BG, Sethi G, et al. Melioidosis: clinical impact and public health threat in the tropics. PLoS Negl Trop Dis. 2017;11(5):e0004738. 28493905 10.1371/journal.pntd.0004738 PMC5426594

[57] Xu H, Wang X, Feng W, et al. The gut microbiota and its interactions with cardiovascular disease. Microb Biotechnol. 2020;13(3):637–656. 31984651 10.1111/1751-7915.13524 PMC7111081

[58] Jin M, Qian Z, Yin J, et al. The role of intestinal microbiota in cardiovascular disease. J Cell Mol Med. 2019;23(4):2343–2350. 30712327 10.1111/jcmm.14195 PMC6433673

